# Building a Healthier Future: A Narrative Review on Early Infant Diagnosis's Role in HIV Prevention

**DOI:** 10.1002/hsr2.70591

**Published:** 2025-03-20

**Authors:** Emmanuel Ifeanyi Obeagu, Getrude Uzoma Obeagu

**Affiliations:** ^1^ Department of Biomedical and Laboratory Science Africa University Mutare Zimbabwe; ^2^ School of Nursing Science Kampala International University Kampala Uganda

**Keywords:** antiretroviral therapy, early infant diagnosis (EID), HIV prevention, HIV screening, infant health, pediatric HIV, public health

## Abstract

**Background and Aims:**

Early infant diagnosis (EID) is a critical intervention in the global fight against pediatric HIV, providing early identification and treatment for HIV‐exposed infants. This narrative review examines the role of EID in reducing vertical transmission, improving health outcomes, and mitigating stigma in communities. The review also explores innovations, challenges, and strategies for optimizing EID programs.

**Methods:**

A comprehensive literature search was conducted across databases such as PubMed, Scopus, and WHO reports to identify relevant studies, guidelines, and program evaluations. Key focus areas included diagnostic techniques, implementation strategies, and community impacts of EID programs, with an emphasis on their integration into broader HIV care frameworks.

**Results:**

EID enables the early detection of HIV in infants, allowing timely initiation of antiretroviral therapy (ART), which reduces morbidity and mortality. It also indirectly prevents postnatal transmission during breastfeeding by lowering viral loads in treated infants. Innovations such as point‐of‐care testing and digital health tools have improved access and efficiency, particularly in resource‐limited settings. EID programs have demonstrated a positive societal impact by raising awareness, reducing stigma, and fostering trust in healthcare systems. However, significant challenges persist, including logistical barriers, infrastructure limitations, and socioeconomic constraints that hinder program effectiveness.

**Conclusion:**

EID is essential for addressing pediatric HIV, contributing to the elimination of vertical transmission and improving outcomes for HIV‐exposed infants. To enhance its impact, stakeholders must prioritize expanding access to diagnostic tools, strengthening healthcare infrastructure, and engaging communities through education and advocacy. EID programs not only save lives but also foster societal shifts towards inclusivity and acceptance, paving the way for a healthier, HIV‐free future.

## 1 Introduction

HIV/AIDS continues to be a significant public health challenge globally, affecting millions of individuals, including infants and young children [1, 2]. Despite advancements in treatment and prevention, the epidemic remains particularly severe in low‐resource settings where access to healthcare and preventive measures is limited. One of the most critical aspects of combating pediatric HIV is preventing mother‐to‐child transmission (MTCT), which can occur during pregnancy, childbirth, or breastfeeding [2]. EID has emerged as a vital strategy in this context, enabling the early detection of HIV in infants and allowing timely intervention that can drastically improve health outcomes and reduce mortality. MTCT is a primary route through which infants acquire HIV. Without intervention, the transmission rate from mother to child can be as high as 45% [3]. However, with effective prevention strategies, including the use of ART during pregnancy, delivery, and breastfeeding, this rate can be reduced to below 5% [4]. EID plays a crucial role in these prevention strategies by ensuring that HIV‐positive infants are identified early and can start ART promptly. Early initiation of ART has been shown to significantly improve survival rates and quality of life for HIV‐positive infants. The importance of EID cannot be overstated. Early detection of HIV in infants is essential because the disease progresses more rapidly in children than in adults. Without treatment, up to 50% of HIV‐positive infants will die by their second birthday, and the majority will not survive past 5 years [4]. EID enables healthcare providers to diagnose HIV within the first few months of life, allowing for early treatment that can prevent these outcomes [5].

Various methods are used for EID, with PCR being the most widely employed due to its high sensitivity and specificity [6]. PCR can detect HIV genetic material in the blood of infants, providing reliable results that guide treatment decisions. DNA‐based tests are also used, particularly in resource‐limited settings, as they are robust and cost‐effective. Rapid diagnostic tests (RDTs) offer preliminary results and are useful in areas with limited laboratory infrastructure. Despite the effectiveness of these methods, their implementation faces significant challenges, particularly in low‐resource settings where healthcare infrastructure may be inadequate. Access to EID services is often limited in low‐resource settings due to several factors [7]. These include a lack of healthcare infrastructure, insufficient training for healthcare workers, and logistical challenges related to sample collection, transportation, and result management. Additionally, the cost of testing and treatment can be prohibitive for many families, impacting the sustainability and reach of EID programs. Addressing these barriers is crucial for expanding access to EID and ensuring that all infants at risk of HIV can benefit from early diagnosis and treatment. Success stories from various regions highlight the potential impact of well‐implemented EID programs. In sub‐Saharan Africa, countries like South Africa and Uganda have made significant strides in reducing pediatric HIV through comprehensive EID strategies [8]. These programs integrate HIV testing with routine maternal and child health services, ensuring that infants born to HIV‐positive mothers are screened and treated promptly. Similarly, in India, national health initiatives have led to increased early detection rates and improved outcomes for HIV‐positive infants, demonstrating the effectiveness of EID in diverse settings. Community engagement efforts can include targeted outreach and education campaigns that address cultural and social barriers to HIV testing and treatment. Involving community leaders and using local languages and media can enhance the effectiveness of these campaigns [9].

## 2 Aim

The aim of early infant diagnosis (EID) is to ensure the early and accurate identification of HIV infection in infants, enabling timely intervention and comprehensive care.

## 3 Justification

The implementation of EID is essential for several compelling reasons, each underscoring the critical role EID plays in addressing the global challenge of pediatric HIV/AIDS [10]. The justification for EID is rooted in its potential to enhance health outcomes for infants, reduce transmission rates, and contribute to broader public health goals. Infants who are HIV‐positive are at a significantly higher risk of rapid disease progression compared to older children and adults [11]. Without early diagnosis and timely intervention, these infants can develop severe HIV‐related illnesses and experience high mortality rates. EID allows for the detection of HIV infection at a stage when the disease is still manageable with ART. By initiating ART early, we can slow the progression of the disease, improve survival rates, and support healthier development. MTCT of HIV remains a major concern in the global fight against HIV/AIDS. MTCT can occur during pregnancy, childbirth, or breastfeeding. Identifying HIV‐positive infants early enables healthcare providers to implement preventive measures and treatment strategies that reduce the risk of MTCT. This includes advising on safe feeding practices, providing ART to both mother and child, and ensuring ongoing care and support. By preventing MTCT, EID helps to break the cycle of HIV transmission and reduces the number of new pediatric HIV cases. Early initiation of ART has been shown to significantly improve health outcomes for HIV‐positive infants. By starting treatment at an early stage, we can prevent or delay the onset of AIDS‐related complications, reduce the frequency of opportunistic infections, and enhance the overall quality of life for these children. Early intervention also supports normal growth and development, allowing affected infants to achieve their full potential. The long‐term economic benefits of EID are substantial. Early diagnosis and treatment reduce the need for more intensive and costly interventions associated with advanced HIV disease.

EID provides valuable data on HIV prevalence and transmission trends among infants. This information is crucial for public health planning and strategy development. By understanding the patterns of HIV transmission and the effectiveness of interventions, public health authorities can tailor their strategies to address the needs of affected populations more effectively. EID programs contribute to broader surveillance efforts, helping to monitor the impact of HIV prevention and treatment initiatives. Implementing EID programs necessitates the strengthening of healthcare infrastructure, including laboratory capabilities, training of healthcare workers, and supply chain management. This investment in health system capacities not only benefits EID but also enhances the overall quality of healthcare services. Improved infrastructure and training contribute to better healthcare delivery across various domains, supporting a more resilient and effective health system. Community education and awareness about the importance of EID play a crucial role in reducing the stigma associated with HIV. By promoting early testing and treatment, EID programs help normalize HIV care and encourage families to seek necessary medical services. Reducing stigma and promoting health‐seeking behaviors are essential for ensuring that all infants at risk of HIV receive timely diagnosis and care. Integrating EID with other maternal and child health services, such as immunizations and nutritional support, provides a comprehensive approach to infant health.

## 4 Review Methodology

### 4.1 Search Strategy

The review process began with the development of a detailed search strategy to identify relevant literature on EID and its role in HIV prevention. Multiple electronic databases were consulted, including PubMed, Scopus, and Google Scholar, to capture a broad range of studies and articles. The search terms included combinations of keywords such as “Early Infant Diagnosis,” “HIV prevention,” “pediatric HIV,” “mother‐to‐child transmission,” and “antiretroviral therapy.”

Inclusion criteria were established to filter the literature. Eligible studies had to meet the following criteria: (1) focus on EID or related interventions, (2) provide empirical data or theoretical analysis relevant to HIV prevention in infants, and (3) be published in peer‐reviewed journals or reputable sources. Studies were excluded if they were not directly related to EID or HIV prevention or if they were not in English due to language limitations.

### 4.2 Selection and Screening

The selection process involved an initial screening of titles and abstracts to identify potentially relevant studies. Full‐text articles were then reviewed to ensure they met the inclusion criteria. This process was conducted by multiple reviewers to enhance reliability and minimize bias. Discrepancies between reviewers were resolved through discussion and consensus.

### 4.3 Importance of Early Infant Diagnosis

EID of HIV is a critical intervention in the fight against pediatric HIV/AIDS [12]. This practice involves testing infants born to HIV‐positive mothers as early as possible to detect the presence of the virus. The importance of EID cannot be overstated, as it plays a crucial role in several areas of public health and individual patient outcomes. EID allows for the early detection of HIV in infants, enabling healthcare providers to initiate ART promptly. This early intervention is crucial because HIV progresses much more rapidly in infants than in adults. Without timely treatment, many HIV‐positive infants develop severe immunodeficiency and are at a high risk of mortality. Studies have shown that starting ART within the first few months of life significantly increases the survival rates of HIV‐positive infants and can help them achieve near‐normal health and development outcomes. Early treatment also reduces the risk of opportunistic infections and other HIV‐related complications [13, 14]. MTCT is the primary way infants contract HIV, occurring during pregnancy, childbirth, or breastfeeding. EID is essential in reducing vertical transmission by identifying infected infants early and ensuring they receive appropriate treatment [15]. Early diagnosis and treatment can lower the viral load in the infant, reducing the likelihood of transmission during breastfeeding. Moreover, EID programs often include measures to ensure that HIV‐positive mothers receive ongoing treatment, which further reduces the risk of transmission to future offspring. EID enables the timely detection of HIV‐infected infants. Without EID, infected infants may remain undiagnosed, allowing viral replication to continue unchecked. Early diagnosis ensures prompt initiation of ART, which can significantly suppress viral replication in the infant. While EID itself does not directly prevent vertical transmission, it intervenes in the progression of the disease, creating conditions that reduce potential postnatal transmission during breastfeeding. ART initiated early in HIV‐positive infants effectively reduces the viral load to undetectable levels. This suppression minimizes the presence of the virus in bodily fluids, including breast milk, reducing the risk of further postnatal transmission. By identifying and treating infected infants early, EID indirectly contributes to preventing secondary transmission during breastfeeding, particularly in settings where mixed feeding or prolonged breastfeeding increases transmission risks. EID also facilitates improved maternal‐infant care. When an infant is identified as HIV‐positive, there is often a concurrent reassessment of maternal treatment adherence and care strategies. This holistic approach can lead to improved maternal viral suppression, further reducing the likelihood of postnatal transmission. EID contributes to vertical transmission prevention not by stopping transmission outright but by breaking the cycle of ongoing viral dissemination through early intervention. The combination of prompt identification, ART initiation, and continuous care disrupts potential postnatal transmission pathways, including during breastfeeding.

EID facilitates continuous monitoring and follow‐up care for HIV‐exposed infants [16]. Early diagnosis ensures that these infants receive regular medical evaluations, including monitoring their response to ART and managing any side effects. This ongoing care is crucial for adjusting treatment regimens as needed and providing comprehensive support to ensure the overall health and well‐being of the child. Regular follow‐up also allows healthcare providers to address any developmental issues early, which is particularly important given the rapid progression of HIV in infants. Implementing EID programs strengthens public health efforts to combat HIV/AIDS [17]. By identifying HIV‐positive infants early, public health officials can collect valuable data on the prevalence and patterns of HIV transmission, which is essential for informing and optimizing public health strategies. Effective EID programs can serve as models for other regions and countries, demonstrating best practices in early diagnosis and treatment. Additionally, EID contributes to reducing the overall burden of HIV/AIDS on healthcare systems by preventing severe HIV‐related illnesses that require intensive and costly treatments. EID programs also have a significant impact on communities and society as a whole. EID programs often include educational campaigns and community outreach to raise awareness about HIV prevention and care. These initiatives help demystify the disease, fostering greater understanding and empathy. By normalizing HIV testing and treatment, particularly for infants, EID programs reduce misconceptions that often fuel stigma. In many communities, HIV stigma is perpetuated by secrecy and fear. EID programs require caregivers and families to engage with healthcare systems openly, helping to break this cycle. When caregivers see infants receiving treatment without judgment, it signals a shift towards more inclusive attitudes, gradually eroding societal stigma. EID programs emphasize accessible and supportive healthcare services. By providing compassionate care for HIV‐exposed infants, these programs build trust between communities and healthcare providers. This trust reduces the fear of discrimination, making individuals more likely to seek testing and treatment for themselves and their families [16]. When communities observe the success of EID programs in ensuring healthy outcomes for HIV‐positive infants, it reinforces the message that HIV is a manageable condition. This realization shifts perceptions from HIV being a “death sentence” to it being a chronic, treatable illness, which can significantly reduce stigma. Stigma is often passed down through generations, with families hiding HIV status or avoiding discussions. EID programs challenge this by focusing on the health of the youngest and most vulnerable, encouraging families to confront HIV openly and positively. This can foster an environment where future generations view HIV with less fear and judgment. EID programs often include maternal health components, emphasizing the role of mothers in ensuring the well‐being of their infants. By highlighting the proactive steps that mothers take through EID, these programs empower women, reducing the blame and stigma they may face for transmitting the virus to their child. On a societal level, successful EID programs can drive policy changes that promote inclusivity and protect the rights of HIV‐positive individuals. For example, reducing stigma in healthcare settings encourages broader antidiscrimination efforts, creating ripple effects across communities [17]. By promoting early diagnosis and treatment, these programs help reduce the stigma associated with HIV. Educating communities about the importance of EID and HIV prevention encourages more people to participate in screening and treatment programs, fostering a more supportive environment for affected families. Empowering communities through education and awareness campaigns can lead to improved health‐seeking behaviors, better adherence to treatment protocols, and overall healthier outcomes for infants and their families. From an economic standpoint, EID can result in substantial cost savings for healthcare systems.

### 4.4 Current Early Infant Diagnosis Methods

EID of HIV is essential for the timely detection and treatment of HIV in infants. Various diagnostic methods are employed to achieve this goal, each with its own advantages and limitations. The primary EID methods include PCR tests, DNA‐based tests, and RDTs.

### 4.5 Polymerase Chain Reaction (PCR) Tests

PCR tests are the most widely used method for EID due to their high sensitivity and specificity [6]. These tests detect the presence of HIV genetic material (RNA or DNA) in an infant's blood sample. The PCR process involves amplifying small amounts of viral genetic material to detectable levels, making it possible to identify HIV even in the early stages of infection.

1. **HIV DNA PCR**: This test detects proviral DNA integrated into the host's genome. It is typically used for early diagnosis within the first few weeks of life, as maternal antibodies can interfere with other testing methods. HIV DNA PCR can identify infection as early as 2 weeks after birth, making it a crucial tool for early intervention.
2. **HIV RNA PCR**: This test measures the amount of viral RNA in the blood, providing information about viral load. While it is not typically used for initial diagnosis due to the presence of maternal RNA, it is valuable for monitoring treatment efficacy and disease progression in diagnosed infants.

### 4.6 DNA‐Based Tests

DNA‐based tests are another reliable method for EID, especially in settings where PCR is not feasible [18]. These tests detect HIV DNA in blood samples, similar to PCR tests, but they often use different amplification techniques that may be more suitable for low‐resource environments.

1. **Loop‐Mediated Isothermal Amplification (LAMP)**: LAMP is a DNA amplification method that is less complex and can be performed at a single temperature, eliminating the need for advanced thermal cyclers used in PCR. This makes LAMP a promising option for use in resource‐limited settings where maintaining precise temperature control is challenging.
2. **Recombinase Polymerase Amplification (RPA)**: RPA is another isothermal amplification method that is rapid and can be performed with minimal equipment. It has shown potential for point‐of‐care (POC) testing, making it accessible for rural and low‐resource areas.

### 4.7 Rapid Diagnostic Tests (RDTs)

RDTs offer a quick and preliminary assessment of HIV status [19]. These tests detect HIV antibodies or antigens in a small blood sample, providing results within minutes. However, their use in infants poses challenges due to the presence of maternal antibodies, which can persist for up to 18 months and lead to false‐positive results.

1. **HIV Antigen Tests**: These tests detect the presence of HIV p24 antigen, which is a part of the virus itself and can be identified earlier than antibodies. While not as sensitive as PCR, antigen tests can be useful in identifying HIV infection during the early stages.
2. **Combination Tests**: Some RDTs are designed to detect both HIV antibodies and p24 antigen, improving their accuracy and reducing the window period during which the virus may go undetected.

### 4.8 Integrated Approaches and Innovations

Innovative approaches are being developed to overcome the limitations of current EID methods and improve accessibility in low‐resource settings [13, 20]. Some promising advancements include:

1. **Dried Blood Spot (DBS) Testing**: DBS involves collecting a small blood sample on filter paper, which can be easily transported to laboratories for testing. This method simplifies sample collection and storage, making it feasible in remote areas.
2. **Point‐of‐Care (POC) Testing**: POC testing devices are being developed to provide rapid and accurate results at the site of patient care. These portable devices can perform PCR or other amplification methods without the need for complex laboratory equipment.
3. **Automated and Digital Platforms**: Automated systems and digital platforms are being integrated into EID workflows to streamline processes, reduce human error, and ensure timely reporting of results. These innovations enhance the efficiency and effectiveness of EID programs.

### 4.9 Challenges and Barriers

Despite the crucial role of EID in the fight against pediatric HIV, several significant challenges and barriers impede its widespread implementation and effectiveness. These obstacles span across various domains, including healthcare infrastructure, financial constraints, logistical issues, and social factors. Addressing these challenges is essential to enhance the reach and impact of EID programs globally [12]. One of the primary challenges in implementing EID programs is the limited healthcare infrastructure in many low‐resource settings. Effective EID requires well‐equipped laboratories capable of performing sophisticated tests like PCR, as well as trained personnel to operate these facilities and manage the testing processes. However, many regions lack the necessary infrastructure and skilled workforce, which hinders the ability to provide accurate and timely HIV testing for infants [21]. The cost of EID, including the procurement of testing kits, reagents, and equipment, as well as the training of healthcare workers, can be prohibitive. In many low‐income countries, healthcare budgets are already stretched thin, and prioritizing EID can be challenging amidst competing health priorities. Additionally, the cost of ART and ongoing care for diagnosed infants adds to the financial burden. Sustainable funding models and international support are crucial to ensure the long‐term viability of EID programs [17].

Collecting samples from remote or rural areas and transporting them to centralized laboratories can be difficult due to poor infrastructure and transportation networks. Delays in sample transport can affect the quality of the samples and the accuracy of the results. Long turnaround times for test results can delay the initiation of ART, which is critical for the health of HIV‐positive infants. Ensuring timely communication of results back to healthcare providers and families is essential for effective intervention. Ensuring a consistent supply of testing kits, reagents, and other necessary materials can be challenging. Stockouts and delays in procurement can disrupt EID services and hinder the timely diagnosis and treatment of infants [12]. The effectiveness of EID programs depends heavily on the capacity and expertise of healthcare workers. In many regions, there is a shortage of trained personnel who can conduct and interpret EID tests accurately. Continuous training and capacity‐building initiatives are needed to equip healthcare workers with the necessary skills and knowledge. Additionally, retention of trained staff in low‐resource settings can be difficult due to low salaries and challenging working conditions [21]. Social and cultural factors can also impede the uptake and effectiveness of EID [22]. Stigma and discrimination associated with HIV can discourage mothers from seeking testing and treatment for themselves and their infants. Misinformation and lack of awareness about HIV and the importance of early diagnosis can further hinder participation in EID programs. Engaging communities through education and awareness campaigns is essential to address these social barriers and promote acceptance and utilization of EID services. Accurate data management and quality assurance are critical components of effective EID programs. However, many regions face challenges in maintaining reliable data systems. Inconsistent data collection, recording, and reporting can lead to gaps in monitoring and evaluating the impact of EID programs. Implementing robust data management systems and quality assurance protocols is necessary to ensure accurate tracking of HIV cases and the effectiveness of interventions. Integrating EID services with other maternal and child health programs can enhance the reach and impact of EID. However, achieving this integration poses challenges, including coordination among different health programs, ensuring the availability of comprehensive care, and addressing potential resource constraints. Integrated approaches require strong leadership, effective planning, and collaboration among various health sectors. Ensuring the sustainability and scalability of EID programs is a significant challenge. Pilot programs and small‐scale initiatives may demonstrate success, but scaling these efforts to reach broader populations requires substantial resources and long‐term commitment. Developing sustainable funding mechanisms, fostering public‐private partnerships, and ensuring government commitment are essential for scaling up EID programs effectively.

## 5 Building a Healthier Future

The global effort to combat pediatric HIV/AIDS through EID is a critical step in building a healthier future for all [23]. EID not only addresses the immediate health needs of HIV‐exposed and HIV‐positive infants but also lays the groundwork for long‐term health improvements and the eventual eradication of pediatric HIV. A robust global health infrastructure is fundamental to the successful implementation of EID programs [24]. Investments in healthcare facilities, particularly in low‐resource settings, are essential to ensure that all infants have access to timely and accurate HIV testing. This includes building and equipping laboratories, training healthcare workers, and developing efficient supply chains for medical supplies. By strengthening health systems, we can create a solid foundation for EID and other critical health interventions. The development and deployment of advanced diagnostic technologies are crucial for expanding the reach of EID programs [25]. Innovations such as POC testing, microfluidic devices, and next‐generation sequencing (NGS) can provide rapid and reliable results, even in remote and resource‐limited areas. These technologies can reduce the turnaround time for HIV test results, enabling faster initiation of antiretroviral therapy (ART) and improving health outcomes for infants.

Integrating EID with other maternal and child health services can enhance the effectiveness and efficiency of health interventions. Combining EID with routine immunizations, antenatal care, and nutrition programs ensures that infants receive comprehensive care and monitoring. This integrated approach not only improves health outcomes for HIV‐positive infants but also strengthens overall health systems and reduces the burden of disease. Community engagement and education are vital components of successful EID programs [26]. Raising awareness about the importance of early HIV diagnosis and treatment can reduce stigma and encourage more families to participate in EID services. Community leaders, health workers, and educators play a crucial role in disseminating information and fostering a supportive environment for affected families. By empowering communities, we can improve health‐seeking behaviors and ensure better health outcomes for all. Sustainable funding and strong policy support are essential for the long‐term success of EID programs. Governments, international organizations, and private sector partners must work together to secure resources and create policies that prioritize EID and other HIV interventions. Sustainable funding models that combine domestic and international resources can ensure the continuity and scalability of EID programs, ultimately leading to the elimination of pediatric HIV. Research efforts should focus on developing new diagnostic tools, optimizing treatment protocols, and identifying best practices for program implementation. Innovation in data management and digital health solutions can also enhance the efficiency and effectiveness of EID programs. A collaborative global response is essential to address the complex challenges of pediatric HIV/AIDS. International cooperation and partnerships can facilitate the sharing of knowledge, resources, and best practices.

## 6 Early Infant Diagnosis's Role in HIV Prevention

EID plays a pivotal role in HIV prevention, particularly in the context of preventing MTCT and reducing the overall burden of HIV/AIDS [27]. By identifying HIV infection in infants at the earliest stages, EID enables timely interventions that can significantly improve health outcomes and prevent the spread of the virus. The role of EID in HIV prevention encompasses several key aspects, including timely initiation of ART, reducing MTCT, enhancing monitoring and follow‐up, and contributing to broader public health efforts. One of the primary benefits of EID is the early initiation of ART in HIV‐positive infants [28]. Infants diagnosed with HIV through EID can begin ART immediately, which is crucial for reducing viral replication, preserving immune function, and improving survival rates. Early treatment has been shown to significantly reduce morbidity and mortality in HIV‐positive infants, enabling them to grow and develop more normally. Furthermore, early ART initiation reduces the likelihood of developing severe opportunistic infections and other HIV‐related complications, thereby improving the quality of life for affected infants. EID is instrumental in reducing MTCT of HIV, which can occur during pregnancy, childbirth, or breastfeeding [29]. By identifying HIV‐positive infants early, healthcare providers can implement measures to reduce the risk of transmission during breastfeeding. This may include advising HIV‐positive mothers on safe feeding practices or providing ART to both mother and child to lower viral load and reduce transmission risk.

EID facilitates continuous monitoring and follow‐up care for HIV‐exposed infants, ensuring they receive comprehensive medical evaluations and support. Regular follow‐up allows healthcare providers to monitor the infant's response to ART, manage any side effects, and adjust treatment regimens as needed. This ongoing care is essential for optimizing health outcomes and ensuring that HIV‐positive infants receive the necessary interventions to thrive. Enhanced monitoring also enables the early detection of any developmental issues, allowing for prompt and appropriate interventions [28]. EID contributes to broader public health efforts by providing valuable data on the prevalence and patterns of HIV transmission. This information is critical for informing and optimizing public health strategies aimed at preventing HIV. Effective EID programs can serve as models for other regions and countries, demonstrating best practices in early diagnosis and treatment. Additionally, EID programs can help to reduce the overall burden of HIV/AIDS on healthcare systems by preventing severe HIV‐related illnesses that require intensive and costly treatments [29]. EID programs have a significant impact on communities and society by reducing the stigma associated with HIV and promoting healthier behaviors. Educating communities about the importance of EID and HIV prevention encourages more people to participate in screening and treatment programs, fostering a more supportive environment for affected families. By reducing the stigma and increasing awareness, EID programs can improve health‐seeking behaviors and ensure better health outcomes for infants and their families. From an economic perspective, EID can lead to substantial cost savings for healthcare systems. Early diagnosis and treatment prevent the progression of HIV to AIDS, reducing the need for more expensive and intensive medical interventions later in life.

## 7 Future Directions

To maximize the impact of EID in the fight against pediatric HIV/AIDS, innovative approaches and strategic initiatives must be adopted [30]. Future directions in EID should focus on enhancing accessibility, improving diagnostic technologies, strengthening healthcare systems, integrating services, and fostering community engagement [31]. Developing cost‐effective, user‐friendly, and accurate diagnostic tools can facilitate broader implementation, particularly in resource‐limited settings. Expanding the use of POC Testing devices that can provide rapid and reliable results at the site of patient care is essential. These portable devices should be easy to use and require minimal infrastructure, allowing for timely diagnosis and treatment initiation in remote and rural areas. NGS technologies offer the potential for highly sensitive and specific detection of HIV, even in low viral load scenarios. Integrating NGS into EID programs could enhance diagnostic accuracy and enable more precise monitoring of viral resistance and mutations. Microfluidic Devices can process small volumes of blood and perform complex diagnostic tests on a chip, making them ideal for resource‐limited settings. Microfluidic technologies could simplify sample processing and reduce the need for extensive laboratory infrastructure.

Robust healthcare systems are fundamental to the success of EID programs [32]. Strengthening these systems involves investing in infrastructure, training healthcare workers, and improving supply chain management. Investing in healthcare infrastructure, such as laboratories and clinics, is crucial for expanding EID coverage. Ensuring that facilities are equipped with the necessary tools and technologies will enhance diagnostic capabilities. Continuous training and capacity‐building initiatives are essential to equip healthcare workers with the skills and knowledge needed to conduct and interpret EID tests accurately. This includes training on new diagnostic technologies and best practices in patient care. Improving supply chain management to ensure a consistent and reliable supply of testing kits, reagents, and medications is critical. Efficient supply chains reduce the risk of stockouts and delays, ensuring uninterrupted EID services. Integrating EID services with other maternal and child health programs can enhance the reach and impact of EID. Combining EID with routine maternal and child health services, such as immunizations and antenatal care, can increase access and uptake. Integrated services provide a comprehensive approach to infant health and ensure that mothers and infants receive continuous care. Incorporating EID with nutrition and growth monitoring programs can address the overall well‐being of HIV‐exposed infants. Early intervention in cases of malnutrition or growth delays can improve health outcomes and support HIV treatment adherence. Community engagement is vital for the success of EID programs. Educating communities about the importance of early HIV diagnosis and treatment helps reduce stigma and encourages participation. Conducting targeted outreach and education campaigns can raise awareness about the benefits of EID and dispel myths and misconceptions about HIV. Using local languages and culturally relevant messages can enhance the effectiveness of these campaigns. Engaging community leaders and influencers can foster trust and acceptance of EID programs. C

Sustained policy and funding support are essential to ensure the long‐term viability and scalability of EID programs [33]. Strong government commitment and leadership are necessary to prioritize EID in national health agendas. Policies that support the integration of EID with other health services and allocate adequate resources are vital. Collaborative efforts between governments, international organizations, nongovernmental organizations (NGOs), and private sector partners can drive innovation and scale successful interventions. International funding and technical assistance can help build capacity and address resource gaps. Developing sustainable funding models that combine domestic resources, international aid, and private sector investments is crucial for the continuity of EID programs. Ensuring predictable and long‐term funding will support program stability and growth. Ongoing research and development are key to advancing EID technologies and strategies. Conducting operational research to identify best practices and effective models for EID implementation can guide policy and program decisions. Evaluating different approaches in various contexts helps tailor interventions to local needs. Investing in the research and development of new diagnostic tests and technologies can address current limitations and improve the accuracy, affordability, and accessibility of EID.

## 8 Conclusion

EID is a cornerstone in preventing and managing pediatric HIV, with profound implications for individuals, families, and communities. The main findings of this review highlight the critical role of EID in identifying HIV‐positive infants early, enabling timely initiation of ART, and significantly reducing morbidity and mortality. Furthermore, EID indirectly contributes to preventing postnatal transmission during breastfeeding by suppressing viral loads in infected infants and improving maternal adherence to treatment protocols. The impact of EID extends beyond clinical outcomes. By promoting awareness and reducing stigma, EID programs empower families, foster trust in healthcare systems, and catalyze shifts in societal attitudes towards HIV. At a broader level, these programs contribute to global efforts to eliminate mother‐to‐child transmission and achieve Sustainable Development Goals (SDGs) related to health and well‐being. In conclusion, strengthening EID programs is essential for building a healthier future. By investing in robust healthcare systems, fostering partnerships, and adopting inclusive policies, stakeholders can ensure that no child is left behind in the fight against HIV. EID is not just a diagnostic tool—it is a transformative intervention that paves the way for a world free from pediatric HIV.

## Author Contributions

**Emmanuel Ifeanyi Obeagu:** conceptualization, methodology, validation, supervision, visualization, resources, writing – original draft, writing – review and editing. **Getrude Uzoma Obeagu:** methodology, validation, visualization, writing – review and editing, writing – original draft.

## Ethics Statement

The authors have nothing to report.

## Conflicts of Interest

The authors declare no conflicts of interest.

### TRANSPARENCY STATEMENT

The lead author Emmanuel Ifeanyi Obeagu affirms that this manuscript is an honest, accurate, and transparent account of the study being reported; that no important aspects of the study have been omitted; and that any discrepancies from the study as planned (and, if relevant, registered) have been explained.

## Data Availability

The authors have nothing to report.
